# Application status and future prospects of the PDX model in lung cancer

**DOI:** 10.3389/fonc.2023.1098581

**Published:** 2023-03-24

**Authors:** Wei Liu, Yishuang Cui, Xuan Zheng, Kunpeng Yu, Guogui Sun

**Affiliations:** Department of Hebei Key Laboratory of Medical-Industrial Integration Precision Medicine, School of Public Health, School of Clinical Medicine, Affiliated Hospital, North China University of Science and Technology, Tangshan, China

**Keywords:** lung cancer, PDX model, pre-clinical research, personalized medicine, co-clinical trials

## Abstract

Lung cancer is one of the most prevalent, fatal, and highly heterogeneous diseases that, seriously threaten human health. Lung cancer is primarily caused by the aberrant expression of multiple genes in the cells. Lung cancer treatment options include surgery, radiation, chemotherapy, targeted therapy, and immunotherapy. In recent decades, significant progress has been made in developing therapeutic agents for lung cancer as well as a biomarker for its early diagnosis. Nonetheless, the alternative applications of traditional pre-clinical models (cell line models) for diagnosis and prognosis prediction are constrained by several factors, including the lack of microenvironment components necessary to affect cancer biology and drug response, and the differences between laboratory and clinical results. The leading reason is that substantial shifts accrued to cell biological behaviors, such as cell proliferative, metastatic, invasive, and gene expression capabilities of different cancer cells after decades of growing indefinitely *in vitro*. Moreover, the introduction of individualized treatment has prompted the development of appropriate experimental models. In recent years, preclinical research on lung cancer has primarily relied on the patient-derived tumor xenograft (PDX) model. The PDX provides stable models with recapitulate characteristics of the parental tumor such as the histopathology and genetic blueprint. Additionally, PDXs offer valuable models for efficacy screening of new cancer drugs, thus, advancing the understanding of tumor biology. Concurrently, with the heightened interest in the PDX models, potential shortcomings have gradually emerged. This review summarizes the significant advantages of PDXs over the previous models, their benefits, potential future uses and interrogating open issues.

## 1 Introduction

Lung cancer, also termed known as bronchogenic carcinoma, is a malignant lung tumor originating in the lung parenchyma or within the bronchi, accounting for approximately 22.7% of malignant tumors. Lung cancer development involves complex and multiple factors and genes. There are more than 50 subtypes of lung cancers broadly classified into small cell lung cancer (SCLC) and non-small cell lung cancer (NSCLC), which account for 15% and 85% of lung cancers, respectively. There are several types of NSCLC, including lung adenocarcinoma (LUAD) (40-50%) and lung squamous cell carcinoma (LUSC) (30%). Other pathological types (5%) include adenosquamous carcinoma (ASC), large cell lung carcinoma (LCLC), and carcinoid tumor (CT) (1–3). The onset of lung cancer is insidious and most clinical patients have distant metastases at the time of definite diagnosis. Regrettably, the overall five-year survival rate of patients with lung cancer is only 17.8% (4, 5). Lung cancer has emerged as the leading cause of cancer-related mortality worldwide. China has experienced an alarming increase in lung cancer incidences and mortality rates yearly (6). Research is needed to identify early markers for lung cancer development to improve treatment outcomes and reduce related mortality. Overall, there is an urgent need to reduce the poor prognosis of lung cancer through accurate early diagnosis and treatment.

There has been rapid and tremendous progress in the development of anti-tumor drugs. In the 1970s, scholars used two-dimensional monolayer cultured cell lines as cancer research models for drug screening. It was later discovered that the established cell lines are selected from specific tumor subsets. Therefore, they do not fully represent the complex clinical intra-tumoral heterogeneity (7). In addition, cell lines lose critical properties after long-term *in vitro* culture. Thus, given the low clinical predictive power of cell lines, most anti-tumor drugs fail in phase III clinical trials. Accordingly, fewer than 5% of candidate drugs are approved for the market (8). In response to the need for effective anti-cancer drugs, the National Cancer Institute (NCI, MD, USA) recommended the PDX models and discontinued the NCI-60 cancer cell line approach (9). The EurOPDX Consortium, containing more than 1500 samples in a PDX bank, also demonstrated that the PDX model is a more feasible tool for *in vitro* research (10, 11). PDX models are developed by implanting cancerous tissue from a patient’s tumor into immunodeficient mice (12). Compared with the cell line models, a PDX model better reflects the structure and microenvironment of the original tumor, and shows less genetic divergence. Besides, PDX models largely retain the primary patient tumor histopathological characteristics and molecular features (13–15). Since PDX model allows several subclones to grow in parallel, it enables them to retain their heterogeneity (10). These advantages make the PDX model a superior platform to facilitate drug development within a shorter period, and identify new targets for cancer therapy. In contrast to cell line models, PDX models can reveal the patterns of tumor evolutionary dynamics under the strong environmental selection pressure and drug resistance mechanisms *in vivo*. Notably, a strong correlation between drug response in PDX models and clinical response (16). Experiments PDXs have revealed accurate therapeutic candidates, minimizing treatment-related toxicity. This review summarizes the salient advantages, recent advances, and gaps in lung cancer precision medicine based on PDX models. Generally, PDX models are key to tackling the precision medicine challenges.

## 2 The pre-clinical models for lung cancer research

To date, a substantial number of the preclinical models for lung cancer research, including traditional lung cancer cell lines (A549, SK-MES-1, HCC827) and genetically engineered mouse models (GEMM), which remain the primary tools for incipient drug development, have been developed (17). Cell line models are rapid and efficient in investigating the potential epigenetic and lung cancer drug treatment mechanisms, attributed to their operation, low cost, high success rate, and high reproducibility. However, the traditional models for lung cancer research face numerous challenges, including cell lines’ loss of critical properties after prolonged *in vitro* culture and the technological inability to interplay with the tumor microenvironment (18). In addition, pre-clinical models may experience gene alterations that cause gain/loss of genetic information and alter the seeding ability and the invasiveness of tumor cells (19). On the other hand, GEMM establishment promotes tumor formation by interfering with specific small molecules, which might influence tumor progression through the alteration of multiple gene loci. For the GEMM model, human-specific immunotherapeutic cannot be tested because mouse biology is not exactly similar to that of humans (20). Therefore, the GEMM model limits preclinical drug research.

Current and ongoing studies based on the PDX models have opened a new avenue for cancer treatment by overcoming the inherent limitations of the traditional models (21–23). The PDX model is the most accurate platform for predicting drug response (24–30). One striking feature of PDX is its ability to overcome cell-line-related limitations. At the cellular level, PDXs retain patients’ heterogeneity and histopathology. Moreover, whole-exome sequencing revealed the high genomic and transcriptional similarity between the PDX model and the primary tumor rearing (31, 32). The PDX model provides clinically relevant pre-clinical tools that simulate the clinical response of patients (33). Also, studies show that PDX models consistently predict response to therapy in patients regardless of passages, supporting the phenotypic stability of these models (34). The PDXs accurately predict cancer treatment response, promoting their use in cancer therapy research. Combined with the molecular omics analysis of PDX models, pathways related to patient tumor biogenesis and development may be discovered. Consequently, in the context of personalized medicine, a PDX model can identify biomarkers that predict cancer treatment response, underlining its usefulness for preclinical cancer research. Thus, PDX can facilitate the identification of lung cancer pathophysiologies and accurate treatment targets. Furthermore, the tumor samples from PDXs provide sufficient materials for cancer-related studies. Research is needed to identify the dynamic changes and genetic characteristics of different tumors to promote the development of targeted therapy and precision medicine (35–37). A PDX model is invaluable for mechanistic research, new drug development, and personalized therapy (38, 39), and relevant for pre-clinical research. High-quality PDX models can have a prognostic value and predict tumor evolution and recurrence probability. They can also reveal potential diagnostic and therapeutic targets, promoting the development of precision medicine (40–42).

## 3 The establishment of the PDX model

### 3.1 Mouse strains used for the PDX model

The mice with an intact immune system will produce robust immune responses to abrogate the implanted tumor tissue. Thus, immunodeficient mice are required to develop the PDX models. Nude CB17-SCID mice, NOD-SCID mice, NOG (NOD/SCID/IL-2γpartial deficiency) mice, and NSG (NOD/SCID/IL-2Rγcomplete deficiency) mice are the common mainstay mouse strains for PDX model establishment. Among them, nude mice are the most commonly used. These mice lack body hair and thymus. Given the absence of the thymus, these mice are T-cell deficient and, thus, cannot induce an adaptive immune response. The nude mouse model was first reported in 1966 by Flanagan (43), which allowed the growth of human cancer cells in mice. Subsequently, the use of CB17-SCID mice and NOD-SCID mice emerged successively. However, due to the high incidence of spontaneous lymphoma, CB17-SCID mice mostly die prematurely (44). In addition, the incidence of immunological leakage in CB17-SCID mice is extremely high.

**Figure 1:**
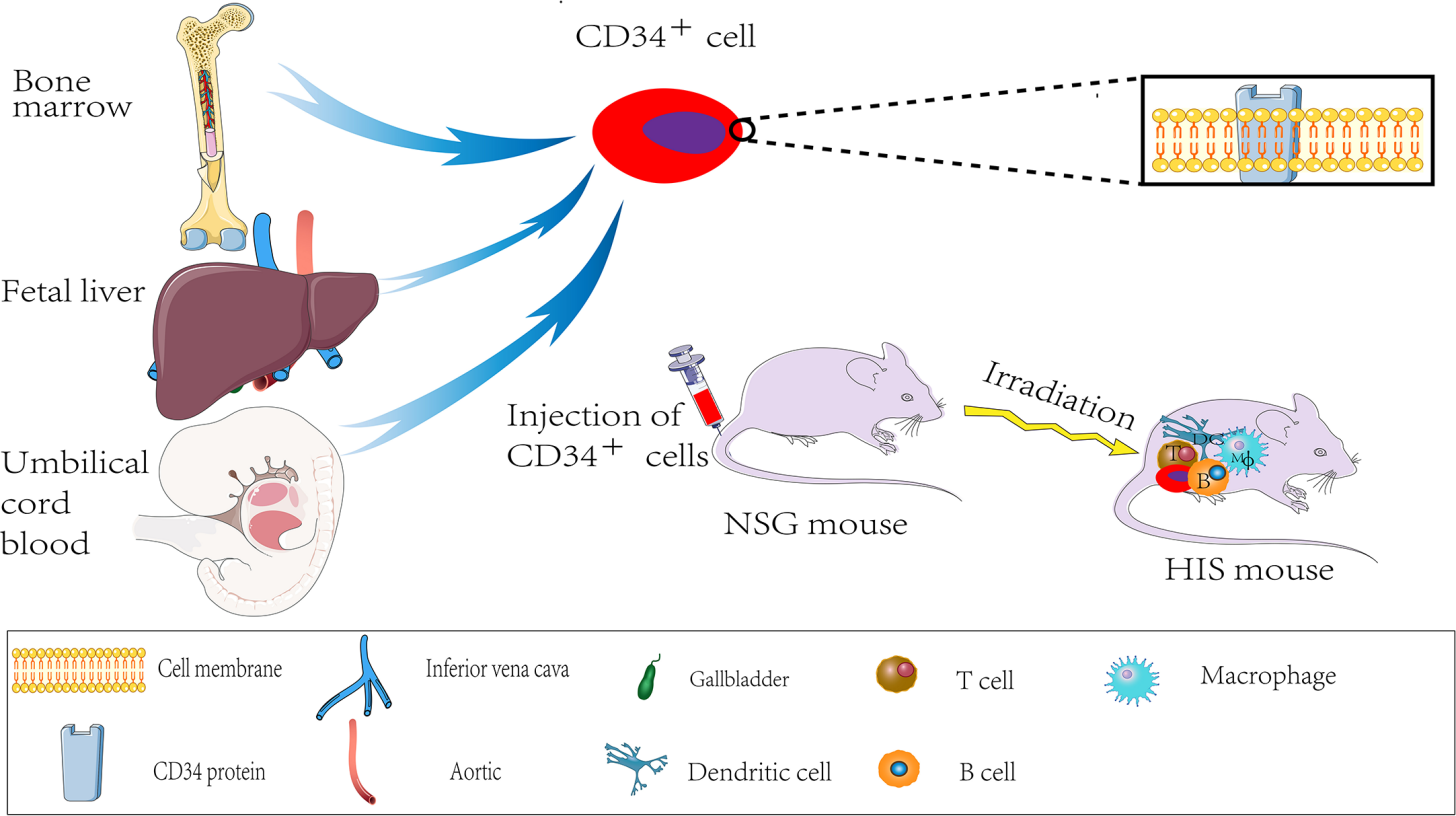
HIS mouse production process.

Similarly, spontaneous lymphoma, in most cases, occurs later in NOD-SCID mice aged around 8.5 months (45). Consequently, neither mouse is suitable for long-term experiments. By the early 2000s, the Central Institute for Experimental Animals (CIEA) developed a severely immunocompromised NOG mouse, significantly improving the survival rate of human cell and tissue transplantation in immunocompromised mice. In 2005, the US Jackson Laboratory cultured NOD/SCID/IL2R gamma (null) mouse, also known as NSG mouse, with a higher transplantation rate and lower tumor graft rejection (46). The NSG mice lack mature T, B, and NK cells for genetic mutations. Thus, the NSG mice are currently the most ideal tumor graft receptors and are less prone to lymphoma development, and have a longer lifespan than other mice (Nude, SCID mice, NOD-SCID mice). The tabular comparison of several immunodeficient mice is shown in **Table 1**. Therefore, the NSG recipient mice offer several advantages for transplantation with human hematopoietic stem cells (46, 47). The NSG mouse is also called “humanized mouse,” or the human immune system (HIS) model because it supports the growth of human hematopoietic stem cells. Thus, it developed an immune system and produced human T cells similar to those of humans (48). The processes of developing the HIS model are sketched in **Figure 1**. The humanized PDX model with patient-matched immune components has several advantages over the alternative models for decoding tumor biology and anti-tumor drug development.

**Table 1:** Comparison between immunized mice.

	Nude	SCID mouse	NOD-scid mouse	NOG/NSG mouse
Mature T cell	–	–	–	–
Mature B cell	++	–	–	–
NK cell	+++	++	+	–
Macrophage	++++	+++	++	+
Dendritic cell	++++	+++	++	+
Incidence of immune leakage	High	High	Lower	Lower
Tumor formation rate	Low	Higher	High	High

Immune function: Hardly (-); Low (+); Medium (++/+++); High (++++).

### 3.2 Implantation tissues and cells and methods

**Figure 2:**
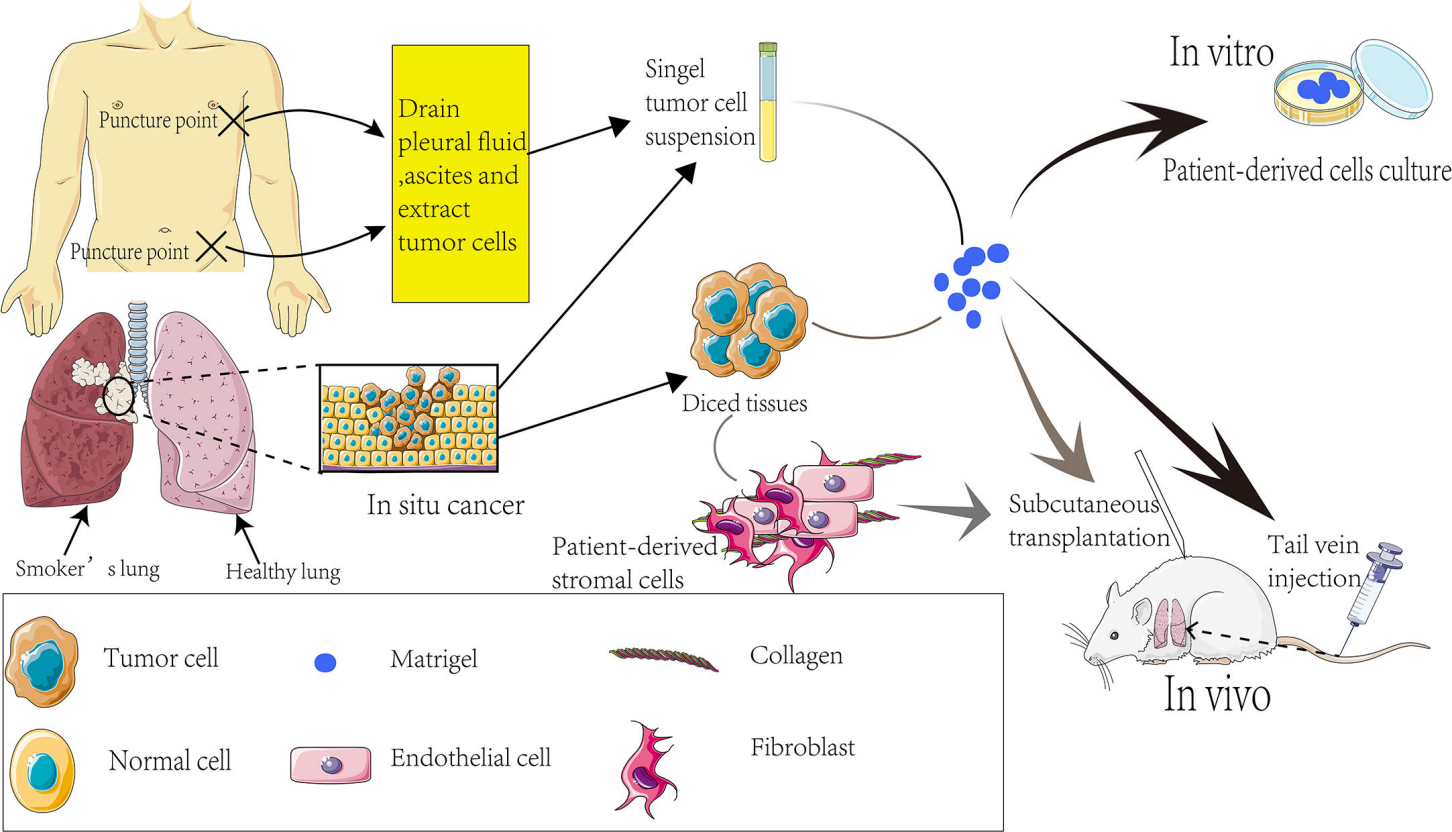
Establishment process of PDX models.

The implanted tissues or cells can be patient biopsies or tumor cells derived from ascites or pleural fluid (**Figure 2**). The implantation tissues comprise small 1-3 mm^3^ clumps or single cell suspensions prepared by digesting small tissue fragments. Co-transplanting with the matrigel increases the engraftment success rates (**Figure 2**). In 1990, Fridman et al. reported that the transplant success rate was significantly higher when lung cancer tissue and matrigel were intertwined and implanted into nude mice (49). To this end, tumor-associated fibroblasts and mesenchymal stem cells provide functional support for tumor progression and development. Furthermore, diversities that may also interfere with the ability of the graft to grow successfully were assessed and compared for ligand-receptor interactions between humans and mice. It was found that some mouse ligands do not activate the corresponding human receptors (50–52).

In brief, proper expression of some human ligands in immunocompromised mice may stimulate graft growth. Ectopic or orthotopic transplantation is traditionally performed *via* subcutaneous and intravenous injection and other forms. The subcutaneous transplantation model is the most extensively used owing to its simple operation and convenient measurement of tumor size. The implantation site is often the back or underarm of mice, which is an excellent model for drug response assessment. Nonetheless, given the limited tumor space, the transplant does not grow in the correct anatomical location. Therefore, this could not be a suitable approach for tumor metastasis research since the tumor does not grow in the correct anatomical location and microenvironment. A differential display analysis is required to compare graft biology and drug response on the *in situ* model and the ectopic model.

Priority should be given to tail vein injection to build an orthotopic transplantation model for tumor metastasis study. The advantage of tail vein injection is that the external environment of *in situ* tumor is closer to the human tumor, providing an experimental basis for subsequent tumor metastasis research (**Figure 2**). Effective transplantation is the nascent basis of the experiment, and many factors can affect the implantation rate. The large vascular bed beneath the renal capsule facilitates the growth of the graft. Xin et al. implanted tumor tissue from NSCLC patients under the renal capsule to obtain an implantation rate of up to 90% (53) and successfully evaluated the sensitivity of patients’ chemotherapy regimen using PDX models. The subcutaneous transplantation success rate is only about 23% (54).

Furthermore, Chen et al. (55) reported that PDX was higher in squamous cell carcinoma, stage II, stage III, and poorly differentiated tumor specimens by following clinically confirmed NSCLC patients for up to 1.5 to 6 years. Additionally, some studies have shown that the implantation rate of PDX tumors after passage increases with the number of passages, and the long-time of tissue *in vitro* is associated with low implantation rates. Moreover, the implantation rate of brain NSCLC metastases is higher than the primary tumor (56), suggesting that the source of the samples potentially affects the implantation rate. Notably, the PDX was not established in the mouse generating human CD20^+^ immune cells. Therefore, adding rituximab during primary tumor implantation may decrease the frequency of lymphoma formation and promote PDX growth.

## 4 Applications of the PDX model for lung cancer research

### 4.1 Immunotherapy

The immune system is responsible for maintaining tumor regression (57). Programmed death ligand 1 (PD-L1) is present on the surface of tumor cells and inhibits the activation and proliferation of T cells, which results in immune escape of tumor cells by specifically binding the programmed death receptor-1 (PD-1) (58, 59). Moreover, T cell depletion is common in people with cancer or chronic infection (60, 61). The T cell depletion is characterized by loss of T cell effector function and continuously increased expression of immune-inhibitory receptors, one of the leading causes of immune disorders in cancer patients. Fortunately, numerous studies have demonstrated the vital role of PD-1 inhibitors in reversing T cell depletion (62–64). In other research, the use of cancer immunotherapy with antibodies, cancer vaccines, and immune checkpoint inhibitors (ICIs) as an emerging and efficient pathway has attracted widespread attention (65–67). Researchers have made unprecedented advances in immunotherapy by utilizing humanized mouse PDX models to accelerate acquired studies of clinically relevant tumors and evaluation of the clinical value of cancer immunotherapy. Humanized mice with human hematopoietic stem cells have been broadly reported as a potent tool in studying targeted PD-1 antibodies (68). Injecting PDX mice with fresh cord blood containing CD34^+^ hematopoietic stem cells to rebuild the immune system significantly shortens the time and regenerates functional, active immune cells. This overrides the delayed tumor transplantation caused by prolonged immune system establishment and the limitation of the incomplete immune system. Immunotherapy provides a new strategy for lung cancer treatment (69, 70). The optimized PDX models are ideal for testing the significant inhibitory effect of the antagonistic PD-1 checkpoint drug-pembrolizumab on lung cancer PDX tumors. Research shows that pembrolizumab-activated human immune cells could effectively limit tumor growth. Targeting cytotoxic T lymphocyte-associated protein 4, PD-1, and PD-L1 using antibodies to block immunomodulatory checkpoints on tumor cells, immune cells, and endothelial cells is an effective NSCLC therapy. Numerous studies have shown that immune checkpoint inhibitors such as PD-L1 and CTLA4 significantly increase the survival rate of advanced NSCLC patients.

Moreover, long patient follow-up studies reveal that the five-year survival rate of NSCLC patients receiving such treatment is as high as 16% (71). The anti-PD-1 drug pembrolizumab is currently approved as an immunotherapy regimen for patients with advanced NSCLC expressing PD-L1 (72–75). Moreover, Nivolumab and Atezolizumab can be used despite PD-L1 expression in NSCLC (76, 77). The studies cited above show great promise in oncology research *via* the use of the PDX model to study and evaluate the necessity of immunotherapy (78).

### 4.2 Drug resistance models for lung cancer

The US Food and Drug Administration has approved more than 20 drugs for the NSCLC treatment, including 14 targeted and immunotherapy drugs. However, the rapid emergence of drug resistance and the diverse resistance mechanisms continue to threaten the ability to treat NSCLC patients. While significant advancements have been made for NSCLC treatment, acquired resistance remains a significant barrier to therapeutic response (79, 80). The genetic alteration uncertainties in patients after treatment is an obstacle to conducting prospective studies. There is an urgent need to identify biomarkers for predicting acquired resistance or poor response of lung cancer to ICIs. The PDX model is ideal for drug resistance studies, including identifying mutations that promote treatment resistance (81). Therefore, the PDX model is critical in the next phase of drug development and reduces the lag in clinical trials. The SCLC patient’s response rate to first-line chemotherapy is about 70%. Nevertheless, most patients experience rapidly acquired resistance within months of treatment, rapid relapse, and poor efficacy of second-line treatment (82, 83). The development of PDX models has been invaluable for investigating acquired drug resistance of tumors.

Previous research reported that EZH2 promotes the development of acquired resistance of PDX by silencing SLFN11. However, after EZH2 knockdown, the PDX tumors’ resistance was suppressed, and the drug efficacy was optimized (84). The exact resistance mechanism of the original tumor was observed in the PDX model of LUAD (85). These studies demonstrate that PDX models accurately predict the patient’s drug response. The widespread uses of ICIs, including PD-1, PD-L1, and CTLA4 antagonistic antibodies, ushered in a new era of cancer immunotherapeutic. However, as PD-1 axis inhibitors become increasingly established in standard treatment options for lung cancer, more patients show resistance to these therapies. Research is needed to identify the mechanisms underlying the acquired resistance of ICIs in lung cancer to overcome the drug resistance of ICIs.

### 4.3 Identification and evaluation of drugs and treatment regimens for lung cancer

**Figure 3:**
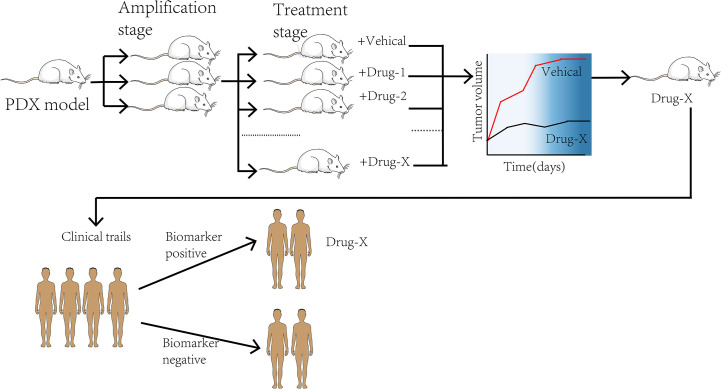
The process of co-clinical trial.

The emergence of the PDX model in drug research development and addressing cancer treatment challenges provides tools for better research. Using the PDX model carrying tumor as the embodiment to evaluate drug efficacy before treatment, it can not only screen for the best anti-cancer treatment but also avoid some adverse drug reactions, which will have a significant impact on patients’ treatment decisions (**Figure 3**). As indicated earlier, the PDX model identifies fewer common microwaves, nanoparticles, gene therapies, and the potential of new anti-cancer drugs for future intervention in tumor development, thus providing a new therapeutic indicator (86). For instance, a PDX model has been used to evaluate three clinical standard protocols for NSCLC patients. The results showed that patients had different sensitivity to the three regimens. Moreover, the patients were not equally sensitive to the same regimen. These findings suggest that the PDX model determines the treatment response and predicts individualized treatment regimens for patients with NSCLC (61). Furthermore, the PDX model predictably addresses the effects of small molecule compounds, antibodies, and microorganisms on lung cancer (87). There are three molecular LUAD subtypes (88–90), with the highly proliferative PP being the most malignant. No validated targeted therapy and immunotherapy have been developed for clinical use against the PP subtype. Therefore, developing specific therapeutic agents will restore hope to patients with the PP subtype. Using nucleic aptamer, Sun et al. found that the leucine-rich PPR-motif containing (LRPPRC) is a specific nucleic aptamer binding protein significantly overexpressed in PP subtype and is strongly linked with the disease stage and patient prognosis. Gossypol acetic acid (GAA) was successfully screened using aptamer-assisted high-throughput methods to specifically bind LRPPRC and cause its degradation in a ubiquitin-proteasome-independent manner. After treatment with GAA, a robust anti-tumor function was shown in the LRPPRC-positive LUAD-PDX models but not in the LRPPRC-negative PDX models. These findings suggest that GAA specifically targeted LRPPRC knockout is a promising therapeutic strategy targeting the PP subtype (91). The above research has entered phase II clinical practice, extending the treatment strategy for lung cancer and highlighting the role of PDX models in developing efficient treatment regimens. Among the functions mentioned earlier, the increase in studies targeting PDXs suggests that PDX models can accurately predict drug response in clinical patients. Therefore, PDX tumor cells can be used for high-throughput screening of anti-cancer therapeutics (92). A study evaluating the use of zebrafish in PDX models reported that transparent zebrafish larvae could visualize individual tumor cells and their response to treatment, acting as a rapid drug screening platform (29). In other words, researchers can fetch the information from the models directly. An intensive drug screening was performed in more than 1,000 PDX models (93). Based on the obtained data, the PDX model has become an essential part of the preclinical screening for new anti-cancer drugs. However, there exist theoretical differences in drug absorption, distribution, and pharmacokinetics between mice and humans, and there may be no natural drug reaction in humans (94, 95). This may result in undesirable consequences after drugs enter clinical trials to determine the pharmacological discrepancies between the two for better future development and testing of anti-tumor drugs.

### 4.4 Co-clinical trials

With the development of individualized, targeted therapies for genotypes, the preclinical studies and clinical trials are conducted simultaneously to elucidate disease mechanisms. In this context, a PDX model can be used as a personalized “Avatar model” for precision medicine in cancer treatment (96). The pre-clinical and clinical trials are conducted simultaneously in tumor patients with specific genetic structures and the corresponding PDX models in co-clinical trials. The trials compare the response between patients and the models in determining the mechanism of action of drugs and finding new targets and biomarkers (97) (**Figure 3**). A group of PDX models directly derived from T2 or T3 stage NSCLC patients was established by Iduna et al. (54). The downregulation of EGFR was observed in the sensitive PDX models after the treatment with cetuximab but not in the resistance models. Iduna confirmed the association of KRAS mutations with the EGFR resistance phenotype, demonstrating that KRAS wild-type status is a clinical biomarker for targeted therapy of NSCLC (54). With such fidelity of the PDX models, it is envisioned that if the pre-clinical studies are performed simultaneously or earlier, the discovery and validation of KRAS mutations as a biomarker for drug resistance will be facilitated. Consequently, such validation linking impactful biomarkers to treatment efficacy will lead to direct clinical effects. The fibroblast growth factor receptor (FGFR) gene is one of the molecular targets of LUSC. As is known to all, dovitinib is a tyrosine kinase inhibitor targeting FGFR. Previous studies have demonstrated that dovitinib has potential anti-tumor capability against tumor cell lines with FGFR1 amplification. Related reports indicated that FGFR1 amplification is associated with poor prognosis in LUSC patients (98). A co-clinical trial concurrent with phase II trials in LUSC patients demonstrated that PDX models accurately replicated patient response to dovitinib. These findings revealed that FGF3 and FGF19 genes were significantly enriched in dovitinib -sensitive tumor cells. In addition, the findings suggest that activation of genes involved in FGFR signaling may be a critical factor in sensitivity to dovitinib (99). The above evidence indicates that the PDX model has strong potential in identifying predictive biomarkers and advancing precision medicine and drug development (100).

## 5 Limitations of the PDX models

Beyond the aforementioned observations, several aspects of the authenticity of the PDX models in oncology research remain to be clarified. For instance, the engraftment of a PDX can cause lymphoma but not the expected tumors. Besides lymphoma, immune cells present in tumors may induce graft versus host disease (GVHD) (101). The tumor samples obtained by means of surgery are limited by the donor site and size, which subtracts from the value of the PDX models constructed that may not cover the overall tumor heterogeneity of patients. Patients with advanced SCLC release a large number of CTCs (102, 103), and because of the random process of CTCs generation in peripheral blood, establishing PDX models through CTCs may be an effective approach to at least partially resolve some problems. Other studies reported some traces of tumor evolution in PDX models, and continuous aged PDX models improved the tumor biology over time (104–106). Surprisingly, alterations in copy number aberrations (CNAs) were found in the models after serial passages, which were somewhat different from the original tumors. The level of CNAs is closely related to drug sensitivity (107) and cancer lethality. When the xenografts are grown to be able for drug screening, the human stromal cells originally present in the original tumor have been replaced by mouse stromal cells, however, some cytokines derived from mice do not exert human-derived cytokines (108), in accordance, some researchers have implanted the patient’s stromal cells together with tumor tissue in mice (**Figure 2**). The cross-reactive between LUSC cells and tumor microenvironment was elucidated in the study by Chen et al. (109). Cancer-associated fibroblasts (CAFs) inhibit SOX2-induced dysplasia and abnormal proliferation of acinar phenotypes in the PDX model-derived TUM622 cell line, and the drug distribution interfered by new mouse stroma can also affect experimental results (109). Studies have shown that the matrigel promotes the survival of mice (48). However, matrigel, a basement membrane substrate derived from EHS mice (110), can cause murine virus infection, hence, its function remains to be clarified, with regard to this facet, using the production of matrigel by ourselves is able to avoid viral contamination to some extent. In addition to the above obvious limitations of the PDX model, the following issues remain to be addressed. Above all, the engraftment failure rate is still high as before (30), it is expensive with little benefit (16). In the next place, the disconnection between the time needed for PDX expansion and treatment (which usually takes 4 to 8 months to build a PDX model) and the rapidity of disease progression in patients. There may be ethical problems under certain conditions (111). Despite the application of “humanized mice” to screen immunomodulators such as vaccines, the challenges that are intrinsic to successfully reconstruct the immune system in mice and maintain a long-term effect. The limitations of the PDX model should be addressed to improve its application in the management of lung cancer.

## 6 Organoids

**Figure 4:**
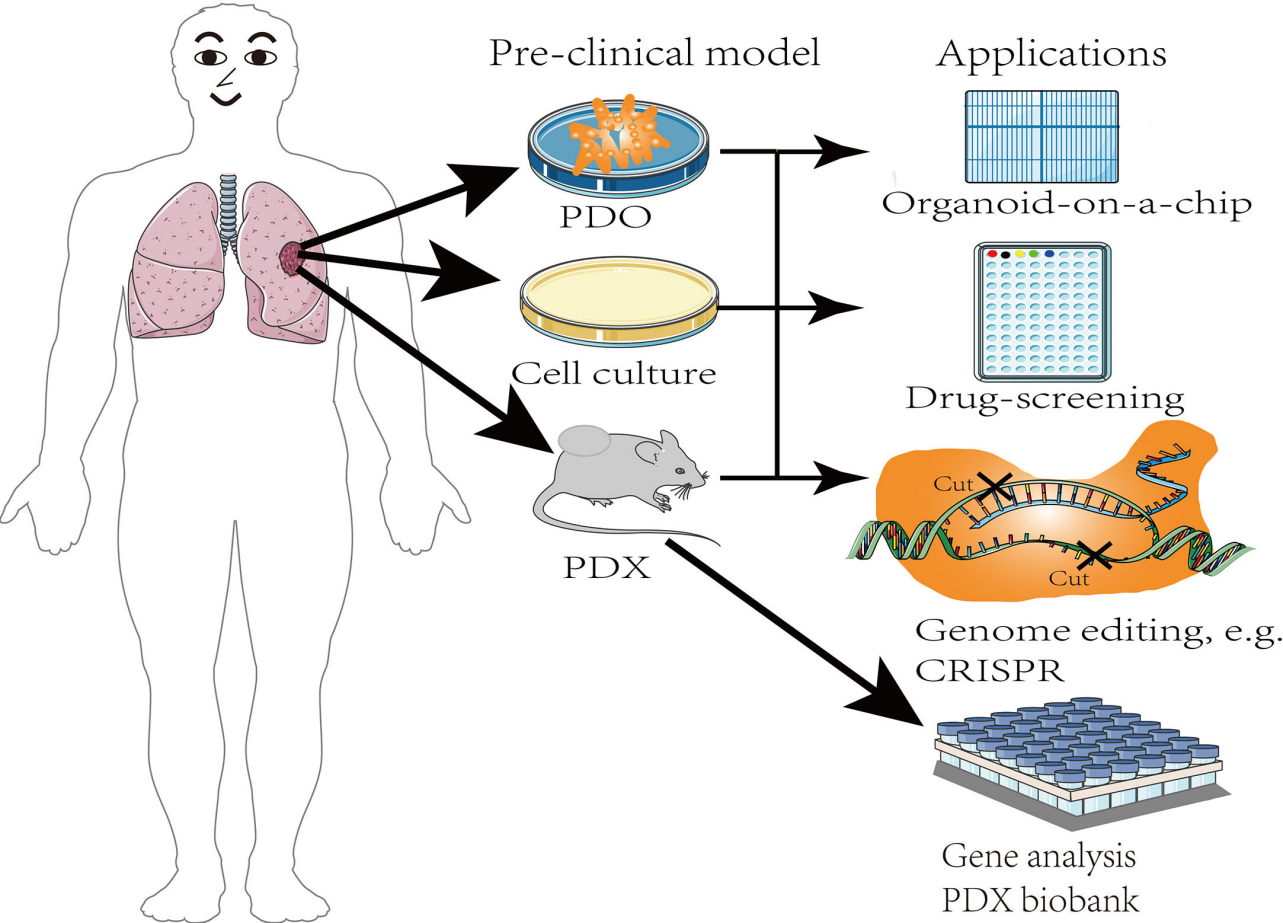
The applications of pre-clinical models.

Given the aforementioned disadvantages of PDX models, stem cells and 3D culture techniques have been used to study organoids. Compared with PDX models, patient-derived organoids shorten the duration of PDX model establishment cycle, increase the transplantation rate, and reduces production costs (112), which contributes to the development of personalized medicine, and are more suitable for large-scale drug screening (**Figure 4**). Notably, organoids combined with other technologies, such as organ chips, 3D printing, and the recently developed approaches that rely upon CRISPR-HOT have improved research on the impact of the tumor microenvironment on tumor development. Organ chips combine organoid technology and microfluidics to provide better control of tumor-related experimental parameters as well as the simulation of the tumor microenvironment during drug screening. Thus, organoids have a greater advantage over the labor-intensive PDX model, and have fewer adverse effects because they gradually replace the human matrix with the mouse matrix at later stages. Furthermore, patient-derived organoid tissue could similarly be used as implant samples for the PDX model. The EVIDENT technology drives the use of microfluidic technology, which allows simultaneous testing of multiple experimental parameters on the same chip, shortens the time course before organoids can be used for drug screening and evaluating patient response to treatment, and reduces experimental errors (113).

## 7 Discussion

A growing body of studies has demonstrated that lung cancer is an evolving cellular ecosystem following Darwinian laws. In light of the intrinsic variability of cancer cells, an early tumor clone produces the offspring of a genetically heterogeneous subclone, which is more malignant. Tumor heterogeneity has a considerable impact on clinical treatment, but its effect on patient response to cancer drugs and how it changes at the genomic and phenotypic level during treatment have not been resolved. These problems highlight the need for pre-clinical research models, increasing interest in developing and applying PDX models in lung cancer research. The PDX model has enabled the production of samples that truly match the patient’s tumor, accurate replication of tumor heterogeneity, progression, and metastatic potential. Considering the growing demand for personalized medicine, the PDX model provides opportunities for developing lung cancer treatment. Using these models for tumor research *in vivo* matches the developmental process of patients’ tumors, which improves drug development for tumor treatment. Although the PDX models meet requirements above, there are several unresolved problems, including implantation methods and selection of mouse strains which influence the implantation rate for lung cancer subtypes with low implantation success rate. Currently, the United States, Europe, and several large pharmaceutical companies such as Novartis have developed systematic PDX resources, a biobank housing thousands of PDX models for lung cancer to solve the precision medical challenges. The PDX samples should be equipped with patients’ clinical data, gene expression patterns, mutation status, drug reactivity, pathological analysis and other data to form a high-precision PDX library and create authoritative resources for personalized treatment of cancer. These PDX biobanks provide good platforms for studying lung cancer and develop new drugs for clinical application.

## Author contributions

GS conceived and designed the, supervised the study, and edited the manuscript. All authors contributed to the article and approved the submitted version.
